# Empiric Antibiotic Therapy for *Staphylococcus aureus* Bacteremia May Not Reduce In-Hospital Mortality: A Retrospective Cohort Study

**DOI:** 10.1371/journal.pone.0011432

**Published:** 2010-07-02

**Authors:** Marin L. Schweizer, Jon P. Furuno, Anthony D. Harris, J. Kristie Johnson, Michelle D. Shardell, Jessina C. McGregor, Kerri A. Thom, George Sakoulas, Eli N. Perencevich

**Affiliations:** 1 Department of Epidemiology and Preventive Medicine, University of Maryland School of Medicine, Baltimore, Maryland, United States of America; 2 Department of Internal Medicine, University of Iowa Carver College of Medicine, Iowa City, Iowa, United States of America; 3 Department of Pathology, University of Maryland School of Medicine, Baltimore, Maryland, United States of America; 4 Department of Pharmacy Practice, College of Pharmacy, Oregon Health and Science University, Portland, Oregon, United States of America; 5 Sharp Memorial Hospital, San Diego, California, United States of America; 6 Iowa City VA Medical Center, Iowa City, Iowa, United States of America; National Institute of Allergy and Infectious Diseases, National Institutes of Health, United States of America

## Abstract

**Background:**

Appropriate empiric therapy, antibiotic therapy with *in vitro* activity to the infecting organism given prior to confirmed culture results, may improve *Staphylococcus aureus* outcomes. We aimed to measure the clinical impact of appropriate empiric antibiotic therapy on mortality, while statistically adjusting for comorbidities, severity of illness and presence of virulence factors in the infecting strain.

**Methodology:**

We conducted a retrospective cohort study of adult patients admitted to a tertiary-care facility from January 1, 2003 to June 30, 2007, who had *S. aureus* bacteremia. Time to appropriate therapy was measured from blood culture collection to the receipt of antibiotics with *in vitro* activity to the infecting organism. Cox proportional hazard models were used to measure the association between receipt of appropriate empiric therapy and in-hospital mortality, statistically adjusting for patient and pathogen characteristics.

**Principal Findings:**

Among 814 admissions, 537 (66%) received appropriate empiric therapy. Those who received appropriate empiric therapy had a higher hazard of 30-day in-hospital mortality (Hazard Ratio (HR): 1.52; 95% confidence interval (CI): 0.99, 2.34). A longer time to appropriate therapy was protective against mortality (HR: 0.79; 95% CI: 0.60, 1.03) except among the healthiest quartile of patients (HR: 1.44; 95% CI: 0.66, 3.15).

**Conclusions/Significance:**

Appropriate empiric therapy was not associated with decreased mortality in patients with *S. aureus* bacteremia except in the least ill patients. Initial broad antibiotic selection may not be widely beneficial.

## Introduction

*Staphylococcus aureus* bacteremia is associated with considerable excess morbidity, mortality, and healthcare costs [1], [2]. The current paradigm for the treatment of suspected invasive bacterial infections, including for *S. aureus* bacteremia, is to prescribe antibiotics as quickly as possible in order to prevent mortality [3]. Yet, optimal treatment strategies for *S. aureus* bacteremia are not precisely defined. Antibiotic therapy with *in vitro* activity to the infecting organism given prior to known culture results, known as appropriate empiric antibiotic therapy, may improve patient outcomes [4]–[10]. On the other hand, over-prescription of antibiotics may lead to an increase in adverse reactions, higher medical costs, and increased antibiotic selection pressure [10].

Previous studies of the association between appropriate antibiotic therapy for *S. aureus* infections and mortality, have demonstrated conflicting results [4]–[16]. The differences in these results may be due to different methods of measuring exposures and outcomes, failure to control for necessary confounders such as severity of illness, or incorrectly statistically adjusting for variables which are part of the causal pathway between infection and mortality [17]–[18]. This study aimed to assess the independent association between receipt of appropriate empiric therapy for *S. aureus* bacteremia and mortality in a large cohort, while statistically adjusting for patient and pathogen characteristics.

## Methods

### Ethics

This study was approved by the institutional review board of the University of Maryland, Baltimore. A waiver of consent was granted given the retrospective nature of the project.

### Study Design and Patient Population

This retrospective cohort study included all adult admissions to University of Maryland Medical Center (UMMC), a tertiary care facility, between January 1, 2003 and June 30, 2007, who had a positive blood culture for *S. aureus*. Each admission was handled as an independent event and therefore patients may have been included in the study more than once. Eligible patients were identified using a relational database that contains medical, pharmaceutical and microbiologic data. These data have been validated in previous studies and have positive and negative predictive values in excess of 99 percent when compared to paper medical records [18]–[23]. Additional variables that were not available in the relational database were collected by a research nurse via chart review.

### Variable Definitions

Antibiotic therapy was defined as appropriate if the *S. aureus* isolate from the blood culture was susceptible to that antibiotic *in vitro*. The timing of appropriate therapy was measured in three different ways. First, empiric antibiotic therapy was defined as receipt of any antibiotic during the period 24 hours before to 24 hours after the culture collection. If the blood culture was collected within 24 hours of hospital admission, the empiric therapy window began at the time of admission. Second, time to appropriate therapy was measured as the time from culture collection to the time appropriate therapy was first received no matter if the appropriate therapy was given empirically or definitively. Third, receipt of any appropriate therapy was a dichotomous variable defined as receipt of appropriate therapy at any time from culture collection to death or discharge.

The outcome of interest, 30-day in-hospital mortality, was measured as mortality occurring in the hospital during the time period from culture collection to 30 days after culture collection. Severity of illness was measured for 24 hours before the time the culture was obtained using the modified Acute Physiology Score (APS). If the blood culture was obtained within 24 hours of hospital admission, APS at the time of admission was calculated. The modified APS is based on the Acute Physiology and Chronic Health Evaluation (APACHE) III score and was measured for all patients in the cohort [24]. Since the APACHE III was originally designed for use in intensive care unit (ICU) patients, the score has been modified by excluding variables that are not applicable to this study population [21], [24], [25]. The Chronic Disease Score, an aggregate measure of comorbid conditions, which has been validated for use in studies on methicillin-resistant *S. aureus* (CDS-MRSA), was calculated using in-patient pharmacy order records [19]. The CDS-MRSA was calculated by assigning a weighted value to medications prescribed for four comorbid conditions (diabetes, peptic ulcers, respiratory illness and kidney disease) within the first 24 hours of admission [19]. Prior history of methicillin-resistant *S. aureus* (MRSA) colonization or infection was measured using infection control documentation of positive surveillance or clinical cultures for MRSA during any previous admission to UMMC.

In 2004, the UMMC clinical laboratory instituted the use of a peptide nucleic acid fluorescence *in situ* hybridization (PNA FISH) (AdvanDx, Woburn, MA) assay which is able to identify the *S. aureus* 16S rRNA from blood cultures approximately three hours after the identification of Gram-positive cocci in clusters [26]. The goal of this assay is to reduce the amount of time necessary to detect *S. aureus* in a blood culture [26]. The institution of the *S. aureus* PNA FISH assay was statistically adjusted for in our analyses because a shorter time to identification of *S. aureus* could reduce the time to appropriate definitive therapy.

### Laboratory methods

All *S. aureus* positive blood cultures during this study period were stored in the clinical laboratory at −80°C. Identification of *Staphylococcus aureus* was determined by gram stain and colony morphology, catalase and coagulase positive reactions or by the *S. aureus* PNA FISH assay. Antimicrobial susceptibility profiles were determined according to Clinical and Laboratory Standards Institute (CLSI) guidelines [27]. Susceptibility testing was performed by the hospital clinical laboratory for erythromycin, clindamycin (including the disk diffusion test for inducible resistance), sulfamethoxazole and trimethroprim, ampicillin and sulbactam, oxacillin, tetracycline, ampicillin, cephalothin, penicillin, rifampin, gentamicin, gatifoxacin, moxifloxacin, and vancomycin. Vancomycin, daptomycin, and linezolid minimum inhibitory concentrations (MICs) were measured using the microdilution Epsilometer test (Etest) (AB BIODISK, Solna, Sweden; bioMérieux, Durham, NC) according to manufacturer's instructions and following CLSI guidelines [27], [28].

Polymerase chain reaction (PCR) was used to determine the presence of the Panton-Valentine leukocidin (PVL) and arginine catabolic mobile element (ACME) genes [29], [30]. The polymorphic X region of the *Staphylococcal* protein A (*spa*) gene was sequenced and typed according to previously described procedures [31], [32]. The USA300 clone was defined as any *S. aureus* isolates that was of *spa*-type motif MBQBLO, PVL-positive, and ACME-positive as previously validated [33].

### Statistical Methods

Bivariate associations were assessed using the chi-square test or Fisher's exact test for categorical variables and the Students t-test or the Wilcoxon Rank Sum test for continuous variables. Cox proportional hazard models were fit to measure the hazard ratios (HRs) and 95% confidence intervals (CIs) for the associations of interest.

All variables that were significant in the bivariable analysis (α<0.1) were included in the initial (full) multivariate Cox proportional hazard model. Variables that were not significantly associated with the outcome (α>0.05) were removed from the full multivariate model in succession. Each of the removed variables was then reinserted into the model to assess whether the variable altered the regression coefficient of the primary exposure variable by greater than 20 percent. If so, that variable was included in the model. Appropriate empiric therapy or time to appropriate therapy was included in each model irrespective of its statistical significance. *A priori* we chose the following variables as biologically important: the main predictor variables (either appropriate empiric therapy or time to appropriate therapy), methicillin resistance, age and severity of illness score. These variables were included in each model irrespective of their statistical significance. In the survival analysis, time to appropriate therapy was treated as a time-varying covariate, which allows for changing hazards over time. Interaction terms were created in order to account for effect modification between variables. In a sub-analysis, the cohort was stratified into quartiles by severity of illness to determine whether severity of illness modified the risk of mortality among patients with bloodstream infections as has been previously demonstrated [34]. All analyses were performed using SAS software (SAS Institute, Cary, NC) version 9.1. Further details of the statistical analysis can be found in Appendix S1.

## Results

Overall, there were 1050 episodes of *S. aureus* bacteremia during the study period. Of these, 814 (78%) had *S. aureus* blood isolates saved and identified for inclusion and molecular analysis. Excluded cases that were missing a *S. aureus* blood isolate did not differ significantly from included cases with regard to methicillin resistance, receipt of appropriate empiric therapy or mortality (data not shown). Among the 814 patients included in the study, 537 (66%) received appropriate empiric therapy and 109 (13%) patients died within 30 days of culture collection.

Patients who received appropriate empiric therapy were more likely to be infected with methicillin-susceptible *S. aureus* (MSSA), to have a polymicrobial infection, to have a higher severity of illness score, to be an injection drug user, to be previously hospitalized in the past year and to have renal disease (p<0.05). Patients who received inappropriate empiric therapy were more likely to have a longer length of stay from admission to culture collection, to be admitted to the ICU before culture collection and to be mechanically ventilated before culture collection (p<0.05) (Table 1). Of those that received appropriate empiric therapy, 75% received vancomycin, 8% received nafcillin or a first generation cephalosporin, 7% received vancomycin and nafcillin or vancomycin and a first generation cephalosporin, 11% received piperacillin/tazobactam, 4% received a third generation cephalosporin, 1% received trimethoprim/sulfamethoxazole, 1% received clindamycin, 1% received daptomycin, 4% received linezolid, and less than 1% received any other antibiotic.

**Table 1:** Characteristics of the Study Population Stratified by Appropriateness of Empiric Therapy.

	Inappropriate Empiric Therapy (n = 277; 34%)	Appropriate Empiric Therapy (n = 537; 66%)	Total (n = 814)	P
Age, mean±SD, years	49±17	49±16	49±16	0.82
Female sex	99 (36%)	210 (39%)	309 (38%)	0.35
Year				0.02
2003	85 (31%)	124 (23%)	209 (26%)	
2004	68 (25%)	136 (25%)	204 (25%)	
2005	47 (17%)	138 (26%)	185(23%)	
2006	56 (20%)	94 (18%)	150 (18%)	
2007^a^	21 (8%)	45 (8%)	66 (8%)	
CDS MRSA, median (IQR)	1.0 (0, 2.2)	1.0 (0, 2.1)	1.0 (0, 2.1)	0.93
Diabetes mellitus	31 (11%)	64 (12%)	95 (12%)	0.79
Renal disease	16 (6%)	57 (11%)	73 (9%)	0.02
AIDS	31 (11%)	74 (14%)	105 (13%)	0.32
Malignancy	24 (9%)	56 (10%)	80 (10%)	0.45
BMI, mean±SD, kg/m^2^	27±7.9	27±9.8	27±9.2	0.53
Modified APS, median, IQR	15 (8, 25)	18 (9, 30)	17.0 (9.0, 28)	<0.01
Time from admission to culture, median, IQR, days	0.94 (0.07, 7.5)	0.17 (0, 3.7)	0.27 (0, 5.0)	<0.01
Prior history of MRSA	33 (12%)	95 (18%)	128 (16%)	0.03
Admitted to ICU prior to culture collection	109 (39%)	138 (26%)	247 (30%)	<0.01
Hospitalized in past year	107 (39%)	249 (46%)	356 (44%)	0.03
Injection Drug Use	71 (26%)	180 (34%)	251 (31%)	0.02
Mechanical ventilation prior to 24 hours before culture collection	70 (25%)	90 (17%)	160 (20%)	<0.01
Infectious disease physician consult±24 hours of culture collection	18 (7%)	57 (11%)	75 (9%)	0.05
Presence of central venous catheter before culture collection	135 (49%)	301 (56%)	436 (54%)	0.05
Polymicrobial Infection	22 (8%)	67 (12%)	89 (11%)	0.05
Methicillin resistance	196 (71%)	292 (54%)	488 (60%)	<0.01
Vancomycin MIC, mean±SD, µg/ml	1.5±0.4	1.5±0.6	1.5±0.6	0.52
USA 300	64 (23%)	126 (23%)	190 (23%)	0.91

Note. Data are no. (%) of admissions, unless otherwise indicated. IQR, interquartile range; MRSA, methicillin-resistant *Staphylococcus aureus*; ICU, intensive care unit; SD, standard deviation; MIC, minimum inhibitory concentration. Polymicrobial infection was defined as more than one microorganism present from the same blood culture.

a Only the first 6 months of 2007 were assessed.

Patients who died within 30 days of hospitalization were more likely to be older, admitted to the ICU before the culture was collected, to have higher severity of illness and comorbidity scores, to have a central venous catheter, and to be mechanically ventilated before culture collection (p<0.05). Patients who survived were more likely to have HIV or AIDS, to have been hospitalized and to receive antibiotics in the past year not counting the index admission, and to be injection drug users (p<0.05). Increased vancomycin MIC and the USA300 clone were not associated with 30-day in-hospital mortality (p = 0.79 and 0.28, respectively) and did not confound the association between appropriate empiric therapy and mortality.

In the bivariate analysis, appropriate empiric therapy was not significantly associated with 30-day in-hospital mortality (unadjusted Hazard Ratio (HR) = 1.52; 95% Confidence Interval [CI]: 0.99, 2.34). The results remained similar when this association was adjusted for severity of illness, methicillin resistance, age, culture collection at admission, polymicrobial infection and ICU admission prior to culture collection using a Cox proportional hazards model (adjusted HR: 1.50; 95% CI: 0.96, 2.35) (Table 2). In order to further adjust for residual confounding due to measured differences between appropriate and inappropriate empiric therapy, a propensity score for the probability of receipt of appropriate empiric therapy was created and added to the final Cox proportional hazard model. As demonstrated in other studies, the addition of a propensity score did not significantly change the hazard ratio or confidence interval for the association between appropriate empiric therapy and mortality (data not shown) [12], [16], [35].

**Table 2:** Components of the final Cox proportional hazard models.

Characteristic	*Adjusted Association between Appropriate Empiric Therapy and 30-day In-hospital Mortality HR (95% CI)*	*Adjusted Association between Receipt of Any Appropriate Therapy and 30-day In-hospital Mortality HR (95% CI)*
Appropriate Empiric Therapy	1.50 (0.96, 2.35)	
Receipt of Any Appropriate Therapy		0.25 (0.10, 0.58)
Severity of Illness a	1.04 (1.03, 1.05)	1.04 (1.03, 1.05)
Methicillin Resistance	1.28 (0.86, 1.92)	1.20 (0.81, 1.79)
Age^b^	1.04 (1.02, 1.05)	1.04 (1.03, 1.05)
Culture collected within 1 hour of admission	1.94 (1.08, 3.50)	2.13 (1.18, 3.84)
Polymicrobial Infection	0.55 (0.30, 1.01)	0.61 (0.33, 1.11)
Admitted to ICU prior to culture collection	2.17 (1.28, 3.68)	2.07 (1.23, 3.50)

a Hazard ratios were calculated as per unit increase of the modified APS.

b Hazard ratios were calculated as per year increase in age.

Among the 774 patients who received appropriate therapy at any time between culture collection and discharge, the median time to appropriate therapy was 0.38 days (interquartile range (IQR): 0.01, 1.22). Time to appropriate therapy was shorter among patients who were hospitalized in the years following when the *S. aureus* PNA FISH assay was instituted (median = 0.34 days; IQR: 0.03, 1.15) compared to those hospitalized in earlier years (median = 0.59 days; IQR: 0.01, 1.43) although this difference only approached statistical significance, p = 0.06. Also, patients infected with MRSA had longer times to appropriate therapy (median = 0.69 days; IQR: 0.07, 1.49) compared to patients infected with MSSA (median = 0.22 days; 0.00, 0.93).

A longer time to appropriate therapy was protective against mortality, although not significantly so (adjusted per-day HR: 0.79; 95% CI: 0.60, 1.03), after statistically adjusting for severity of illness, age, methicillin resistance, prior history of MRSA, culture collection after the *S. aureus* PNA FISH assay was instituted, and admission to the ICU before culture collection (Table 3). When the cohort was stratified into quartiles by severity of illness, a longer time to appropriate therapy was a risk factor for mortality among the healthiest quartile of patients but was protective against mortality for the other quartiles (Table 3).

**Table 3:** Survival Analysis Models of Adjusted Association between Time to Appropriate Therapy and 30-day In-hospital Mortality Stratified by Severity of Illness.

Characteristic	Total Cohort (N = 814)	Quartile 1 Modified APS<9 (n = 195) HR (95% CI)	Quartile 2 Modified APS ≥9 and <17 (n = 208) HR (95% CI)	Quartile 3 Modified APS ≥17 and <28 (n = 195) HR (95% CI)	Quartile 4 Modified APS≥28 (n = 216) HR (95% CI)
Time to Appropriate Therapy	0.79 (0.60, 1.03)	1.44 (0.66, 3.15)	0.71 (0.29, 1.74)	0.58 (0.29, 1.15)	0.71 (0.49, 1.02)
Methicillin Resistance	1.32 (0.87, 2.00)	4.62 (0.51, 41.91)	2.19 (0.49, 9.86)	0.61 (0.26, 1.41)	1.55 (0.89, 2.69)
Age	1.04 (1.03, 1.05)	1.04 (0.99, 1.09)	1.03 (0.99, 1.07)	1.02 (0.99, 1.05)	1.03 (1.02, 1.05)
Admitted to ICU prior to culture collection	1.49 (0.98, 2.27)	8.70 (2.01, 37.72)	3.81 (0.89, 16.39)	1.98 (0.83, 4.74)	1.12 (0.67, 1.87)
Time *S. aureus* PNA FISH Assay in Use	0.80 (0.54, 1.20)	6.40 (0.98, 41.83)	2.85 (0.69, 11.71)	0.33 (0.13, 0.85)	0.80 (0.48, 1.35)
Modified APS	1.04 (1.03, 1.05)	–	–	–	–

Before death or discharge, 774 (95%) patients received any appropriate (empiric or definitive) therapy and 40 (5%) never received appropriate therapy. Thirteen percent of the patients who received any appropriate therapy and 18% of the patients who never received appropriate therapy died within 30 days of hospitalization (p = 0.43). Those that received any appropriate therapy were significantly less likely to die (adjusted HR: 0.25; 95% CI: 0.10, 0.58) compared to those who never received appropriate therapy after statistically adjusting for severity of illness, methicillin resistance, age, culture collected within an hour of admission, polymicrobial infection and admission to the ICU prior to culture collection (Table 2).

Stratified analyses were performed across numerous clinically significant strata but did not change the associations between appropriate empiric therapy or time to appropriate therapy and mortality. For example, when the association between appropriate empiric therapy and mortality was stratified by methicillin resistance, the measures of effect remained similar (relative risk (RR) among MRSA: 1.68; RR among MSSA: 1.41; Breslow-Day Test for Homogeneity p-value = 0.75). The measures of effect for the association between appropriate empiric therapy and mortality did not change when stratified by presence of diabetes (RR among diabetic patients: 2.26, RR among patients without diabetes: 1.33; Breslow-Day Test for Homogeneity p-value = 0.37), or surgery on the index admission (RR for surgical patients: 2.71, RR for non-surgical patients: 1.40; Breslow-Day Test for Homogeneity p-value = 0.52). Additionally, when we stratified the cohort by ICU admission, a longer time to appropriate therapy was protective against mortality among both ICU and non-ICU patients (ICU HR: 0.91; non-ICU HR: 0.74) except among the healthiest quartile of patients (ICU HR: 1.23; non-ICU HR: 2.28).

Sub-cohort analyses were also performed. When we excluded patients who only received vancomycin empirically (n = 305), we found a similar association between receipt of appropriate empiric therapy and mortality (adjusted HR: 1.03; 95% CI: 0.53, 1.97). Also, when we limited the cohort to include only the first admission from each patient (n = 761) and repeated the Cox proportional hazard analyses, the magnitude and statistical significance of the associations of interest remained similar.

## Discussion

In this large cohort of patients with *S. aureus* bacteremia, appropriate empiric therapy and time to appropriate therapy were not associated with decreased mortality except in those patients with the lowest underlying severity of illness score. These results are consistent with previous studies which did not find significant independent associations between appropriate empiric therapy and mortality [12]–[16]. Our study differs from these publications in that our study had a much larger patient population and we incorporated characteristics of the infecting pathogen, such as antibiotic minimum inhibitory concentrations and presence of the USA300 clone, into the analysis. Two of these smaller studies assessed the association between inappropriate empiric therapy and 30-day mortality and found that inappropriate empiric therapy was protective, although not significantly so, against mortality [15], [16]. A potential explanation for these results is that a delay in the receipt of appropriate antibiotic therapy may not have affected the progression of the infection among the patients that died [12], [15]. This hypothesis is supported by the protective effect of appropriate therapy given at any time against mortality. This may indicate that the eventual receipt of appropriate antibiotics is protective against mortality, but the timing of the receipt of appropriate antibiotics in the initial 24 hours is less important.

Prior study results of a significant association between appropriate antibiotic therapy and mortality may be due to different methods of measuring exposures and outcomes, failure to control for necessary confounders such as severity of illness, or incorrectly statistically adjusting for variables which are part of the causal pathway between infection and mortality. Studies by Lodise et al., Romero-Vivas et al., and Soriano et al. did not differentiate between empiric and definitive antibiotic therapy [4], [6], [8]. Therefore, the results of those studies are similar to our finding that patients who ever received appropriate antibiotic therapy were less likely to die compared to patients who never received appropriate antibiotics. Studies by Robinson et al., Leibovici et al., and Shorr et al. found that appropriate empiric therapy was associated with mortality in bivariate analyses but did not control for variables such as age, severity of illness or underlying comorbidities which could potentially confound the association between appropriate empiric therapy and mortality [7], [10], [11]. Finally, studies by Schramm et al., Soriano et al., and Gomez et al. controlled for shock, which is part of the causal pathway between *S. aureus* bacteremia and mortality [5], [8], [9]. Differences in results could also be due to differences in study populations. Some studies only assessed appropriate empiric therapy for MRSA, some included all sterile sites of infection and all studies were from geographically diverse areas which may exhibit differences in antibiotic prescribing, antibiotic resistance trends and differing patient populations.

Our finding that a longer time to appropriate therapy was a risk factor for mortality among the healthiest quartile of patients but was protective against mortality for the other quartiles indicates that efforts to prescribe broad-spectrum antibiotics to severely ill patients may not be beneficial. However, these associations may be affected by residual confounding by indication. Confounding by indication occurs when a characteristic is an indication for the treatment of interest and is a risk factor for the outcome of interest [36]. As our study showed, severely-ill patients were more likely to both receive appropriate antibiotics early (most likely in the form of broad-spectrum antibiotics) and to die compared to less severely ill patients. Thus, despite our attempts to control for severity of illness, residual severity of illness may have confounded the relationship between time to appropriate therapy and mortality.

Alternatively, the effect of time to appropriate antibiotic therapy on mortality may be greater among less severely ill patients compared to more severely ill patients. This is supported by Kim et al. in a study that compared mortality hazards among patients with and without bloodstream infections, stratified by severity of illness. That study found a significant difference in mortality hazards among patients with lower severity of illness scores on admission (HR: 2.42; 95% CI: 1.70, 3.44) but no difference when this association was assessed among more severely ill patients (HR: 0.96; 95% CI: 0.76, 1.23). Kim hypothesized that a bloodstream infection may be of greater consequence among the less severely ill, while the addition of a bloodstream infection to the number of life-threatening conditions among severely ill patients does not considerably decrease their probability of survival [34].

Currently, vancomycin appears to be the drug of choice for appropriate empiric therapy. However, studies have shown that vancomycin may be associated with increased *S. aureus* treatment failure compared to other antibiotics [37], [38]. In this study, the exclusion of patients who only received vancomycin empirically did not change the association between appropriate empiric therapy and mortality.

A limitation of this study is that it was performed at a single center and therefore these results may not be generalizable outside of this patient population. This study was also limited by its observational nature. However, a randomized control trial is not feasible in this situation because it would be unethical to randomize patients to receive inappropriate empiric therapy. Lastly, we were unable to assess whether mortality was due to the *S. aureus* bacteremia and not other causes. Thus, our use of 30-day in-hospital mortality may be an over-estimate of infection-related mortality. However, 30-day in-hospital mortality has been used routinely as an outcome in other *S. aureus* studies [7], [39].

In summary, choice of initial antibiotic therapy must weigh the expected clinical benefits of the individual patient against the public health implication of overuse of antibiotics. Appropriate empiric therapy may reduce adverse outcomes in healthier patients, but initial broad empiric coverage for *S. aureus* bacteremia among severely ill patients may not be beneficial. Therefore, future efforts should continue to identify casual factors associated with increased mortality in patients with *S. aureus* bacteremia.

## Supporting Information

Appendix S1 Detailed Statistical Analysis. Detailed Description of Statistical Analysis
(0.03 MB DOC)
