# Supplementary material for: Empiric Antibiotic Therapy for Staphylococcus aureus Bacteremia May Not Reduce In-Hospital Mortality: A Retrospective Cohort Study
Source: PLoS One. 2010 Jul 2;5(7):e11432. doi: 10.1371/journal.pone.0011432 (PMC2896397; doi:10.1371/journal.pone.0011432)
Supplement: Appendix S1 — Detailed Statistical Analysis. Detailed Description of Statistical Analysis (0.03 MB DOC) [file pone.0011432.s001.doc]

***Appendix: Detailed Statistical analysis***

Univariate analysis was done to evaluate the distribution of all variables. Bivariate analysis was used to measure the correlation between coviariates (patient and pathogen characteristics) and the associations between covariates and main predictor variables, as well as the association between covariates and death. The main predictor variables were appropriate empiric therapy, time to appropriate therapy or receipt of appropriate antibiotics at any time. The correlation between normally-distributed variables was measured using the Pearson correlation coefficient. The correlation between skewed variables was measured using the Spearman correlation coefficient. No two variables were highly correlated (r>|0.7|), thus all could be included in the same model. Bivariate associations were assessed using the chi-square test or Fisher’s exact test for categorical variables and the Student t test or the Wilcoxon Rank Sum test for continuous variables. The Breslow-Day test was used to test whether the potential effect modifiers chosen *a priori* modified the relationship between the main predictor variables and mortality. The potential effect modifiers chosen *a priori* were vancomycin susceptibility, methicillin susceptibility (MSSA vs. MRSA), and severity of illness. When a variable was suspected of modifying the relationship between appropriate empiric therapy and mortality, models were created that were stratified by that variable.

Survival analysis was performed to assess the association between the main predictor variables and 30-day in-hospital mortality. Patients were censored upon hospital discharge. Cox proportional hazard models were created to measure the hazard ratios (HRs) and 95% confidence intervals (CIs) for three separate associations:

1. The association between receipt of appropriate empiric therapy for *S. aureus* bacteremia and 30-day in-hospital mortality
2. The association between time to appropriate empiric therapy for *S. aureus* bacteremia and 30-day in-hospital mortality
3. The association between receipt of appropriate antibiotics for *S. aureus* bacteremia at any time and 30-day in-hospital mortality.

All variables that were significantly (α=0.1) associated with the main predictor variable (see Table 1) or the outcome (30-day in-hospital mortality) in the bivariate analysis or considered biologically important were included in the initial (full) multivariate Cox proportional hazard model. The main predictor variable (appropriate empiric therapy, time to appropriate therapy or receipt of appropriate antibiotics at any time) was included in the model irrespective of its statistical significance. *A priori* we chose the following variables as biologically important: methicillin resistance, age and severity of illness score. These variables were included in each model irrespective of their statistical significance.

All variables that were significant in the bivariable analysis (α<0.1) were included in the initial (full) multivariate Cox proportional hazard model. Variables that were not significantly associated with the outcome (α>0.05) were removed from the full multivariate model in succession unless they were considered biologically important *a priori*. Each of the removed variables was then reinserted into the model to assess whether the variable altered the regression coefficient of the primary exposure variable by greater than 20 percent. If so, that variable was included in the model. The proportional hazards assumption, that the effect of a variable is constant over time, was tested for each variable in the final model by assessing the interaction of the variable with a function of time. These interaction terms were included in the final model one at a time to assess their significance. In the Cox proportional hazard model of the association between time to appropriate therapy and 30-day in-hospital mortality, time to appropriate therapy was treated as a time-varying covariate which allows for changing hazards over time.
